# Weinreb Amides as Directing Groups for Transition Metal-Catalyzed C-H Functionalizations

**DOI:** 10.3390/molecules24050830

**Published:** 2019-02-26

**Authors:** Jagadeesh Kalepu, Lukasz T. Pilarski

**Affiliations:** Department of Chemistry-BMC, Uppsala University, BOX 576, 75-123 Uppsala, Sweden; jagadeesh.kalepu@kemi.uu.se

**Keywords:** C-H functionalization, directing groups, amides, transition metals, catalysis

## Abstract

Weinreb amides are a privileged, multi-functional group with well-established utility in classical synthesis. Recently, several studies have demonstrated the use of Weinreb amides as interesting substrates in transition metal-catalyzed C-H functionalization reactions. Herein, we review this part of the literature, including the metal catalysts, transformations explored so far and specific insights from mechanistic studies.

## 1. Introduction

The pursuit of efficient methods for the direct, catalytic substitution of otherwise inert C-H bonds in organic molecules has become a major area of research focus in recent years [1]. Tremendous advances have been made in the development of previously impossible transformations [2,3,4,5], mechanistic understanding [6,7,8,9], milder and safer protocols [10], and selectivity [11,12,13,14]. The ubiquity of C-H bonds in organic molecules makes their regioselective activation and substitution particularly attractive, but also challenging. To this end, the use of directing groups–parts of an organic substrate that can coordinate to and position a metal center over the desired C-H bond–has met with enormous success [15]. The use of *ortho*-directing groups especially has provided the basis of many new homogeneous catalytic C-H functionalization reactions [16]. Directing groups able to deliver *meta* [17,18] and *para* [19] selectivity in C-H functionalization catalysis have also been described, although the generality of that approach is still some way off in the future.

Ideally, directing groups should not require separate and/or laborious installation/removal or otherwise be inutile in later steps of a synthesis. It is preferable that they should either be part of the desired target compound or that they could be converted to another useful group once their role during the C-H functionalization step is over. A plethora of strategies to realize the latter objective has been pursued. The invention of various removable or modifiable [20,21,22], traceless [23], or otherwise transient [24,25,26,27,28,29] directing groups has formed a sizeable category in and of itself within the field of C-H functionalization catalysis.

Amides are an incomparably important class of compounds. The development of methods for their selective synthesis and derivatization is, therefore, a key pursuit in synthetic methodology. That the amide group can serve as a ligand for transition metals enables various new approaches to this via catalytic C-H functionalization [30]. Most commonly, the amide group has been called upon to direct the catalytic substitution of neighboring Ar-H bonds but, as discussed below, they also enable C(sp^3^)-H bond manipulation.

**Figure 1:**
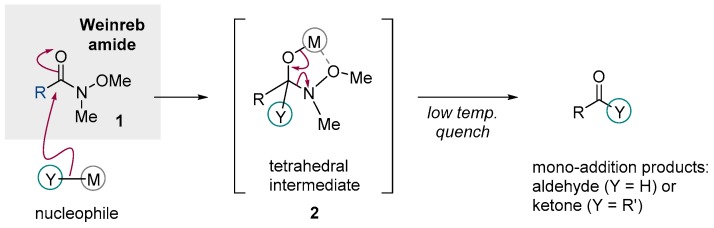
The ‘textbook’ application of Weinreb amides: generation of mono-addition products resulting from nucleophilic attack on their carbonyl groups.

*N*-methoxy-*N*-methyl amides (**1**, Figure 1), or Weinreb amides [31], are a valuable branch of the amide family. They provide the ‘textbook’ route to mono-addition products (especially ketones and aldehydes) via nucleophilic attack on the carbonyl group. Such attacks give rise to stable five-membered tetrahedral cyclic intermediates (**2**) to which a second addition (“over-addition”) is precluded. Weinreb amides may be prepared with ease from carboxylic acids or their chlorides, esters, aldehydes or ketones [32].

**Figure 2:**
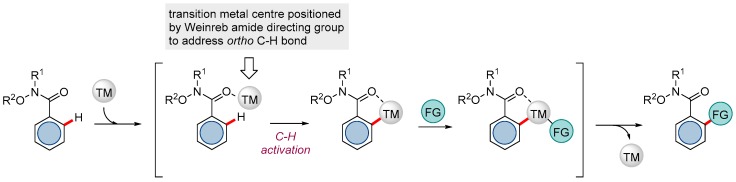
A generic representation of a Weinreb amide-directed catalytic C-H functionalization (TM = transition metal).

That Weinreb amides can also steer the regioselectivity of transition metal-catalyzed C-H functionalizations (Figure 2) qualifies them as noteworthy multi-functional directing groups. Remarkably, and despite the various synthetic advantages of this (for example that it can obviate the need for lithiation strategies and make available previously impossible reactions), Weinreb amides have only recently attracted attention as substrates for C-H functionalization. In part, this is due to the amide oxygen’s weaker coordination ability to most transition metal centers. The latter has presented a challenge to the development of their use in C-H functionalization reactions, although many carbonyl-directed reactions are now known [33].

This review describes progress in the use of Weinreb amides as directing groups in catalytic C-H functionalization. A variety of reactions falls under this category. We have chosen to group these according to the transition metal center responsible for the C-H functionalization catalysis, rather than the overall transformation, in order to maximize the ease of comparison between researchers working in similar areas, and to track the different rates at which progress has occurred and insights in to the underlying mechanisms. Overwhelmingly, the focus of the studies reviewed herein falls on the utility of Weinreb amides as directing groups for the C-H functionalization step. Therefore, whilst several publications describe subsequent manipulation of the Weinreb amide group, this is usually to illustrate the possibility, rather than a key development. We have opted therefore not to include many examples of the latter; we take the possibility of *a posteriori* conversion of Weinreb amides using conventional approaches (e.g., to ketones or aldehydes), for the most part, to be a safe assumption.

## 2. Ru-catalyzed Reactions

**Scheme 1:**
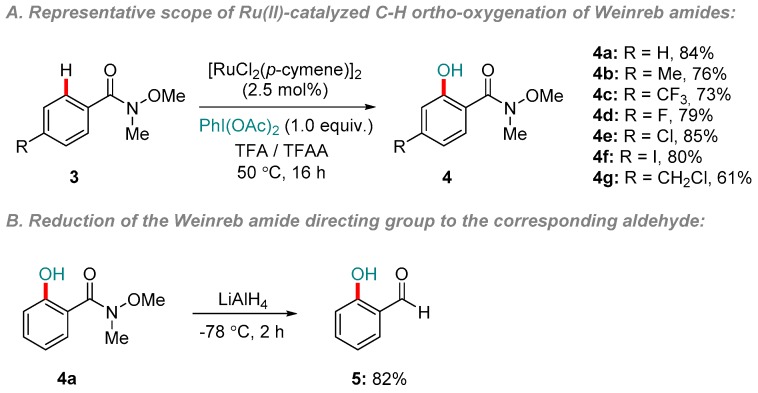
Ru(II)-catalyzed C-H oxidation of arenes directed by a Weinreb amide group. (**a**) Representative scope of the reaction with respect to arene substituents; (**b**) Reduction of the Weinreb amide to reveal aldehyde functionality.

The versatility of Ru, as well as its considerably lower price compared to other 2nd and 3rd-row transition metals, make it an appealing candidate around which to develop economical C-H functionalization methodology [34,35]. Moreover, considerable advances have been made in Ru-catalyzed C-H functionalization directed by weakly coordinating groups, including amides [33]. In 2013, Ackermann and co-workers described the Weinreb amide-directed C-H *ortho*-oxygenation of arenes **3** using a Ru(II)-based system [36]. [RuCl_2_(*p*-cymene)]_2_ served as the catalyst precursor and PhI(OAc)_2_ as the most effective oxidant. Representative results from the study are shown in Scheme 1a.

The reaction showed a high selectivity for mono-oxygenated products **4**, a preference for electron-rich substrates in competition experiments, and a kinetic isotope effect (KIE) of *k*_H_/*k*_D_ = 3.0 [6]. The authors proposed that an irreversible C-H activation event was a key step *en route* to the products. It is notable that the *N*-alkyl substituent could also be varied substantially without any loss of reaction efficiency. The Weinreb amide group could be reduced in high yield (**4a** to **5**) to reveal the corresponding aldehyde (Scheme 1b). A single Weinreb amide substrate was also shown to work as part of a study by Jeganmohan and co-workers on the *ortho*-directed C-H benzoyloxylation of various amides. The reaction system closely resembled that reported above by Ackermann, except for the use of (NH_4_)S_2_O_8_ as the terminal oxidant, higher temperatures and use of 1,2-dichloroethane as solvent [37].

**Scheme 2:**
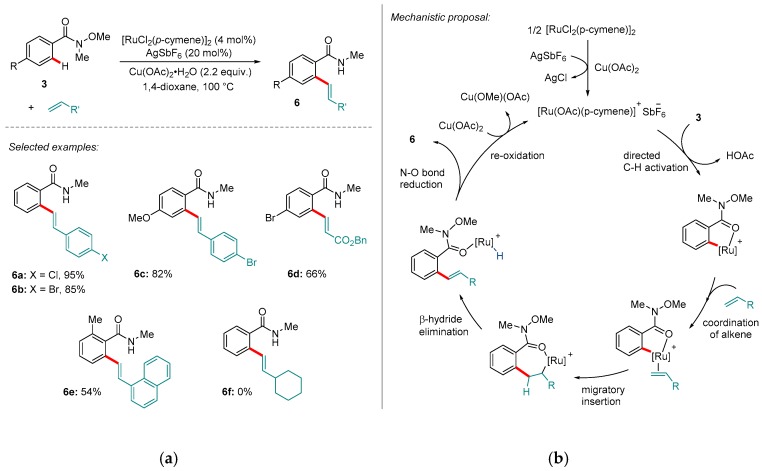
Ru(II)-catalyzed oxidative C-H *ortho*-alkenylation of Weinreb amides; selected products and proposed mechanism.

Subsequently, Das and Kapur produced a report demonstrating the Ru-catalyzed olefination of various amides, including Weinreb amide-decorated arenes [38]. The protocol closely resembles the Pd-catalyzed oxidative Heck reaction [39] (also known as the Fujiwara-Moritani reaction) and other related Ru-catalyzed C-H alkenylations [34]. However, for the majority of their entries (selected examples are shown in Scheme 2a), the authors observed cleavage of the Weinreb amide N-O bond; *N*-methyl amides were obtained as the main products. It is instructive to consider the proposed mechanism, an adapted version of which is shown in Scheme 2b. Presumably, the N-O bond serves as an oxidant with Cu(OAc)_2_∙H_2_O as the carboxylate source to facilitate repeated C-H functionalization [40] The use of similar “internal oxidant” strategies in C-H functionalization, including using N-methoxy amides, has been recently reviewed by Cui and co-workers [41]. Exceptions wherein the N-O bond was preserved presumably resulted from Cu(II) (or Cu(III) species arising via disproportionation) outcompeting N-O as an internal oxidant. The importance of such examples is under-appreciated, in our view. That an exogenous oxidant may divert reactivity away from damaging a group under otherwise identical conditions is a key aspect of modulating functional group tolerance; a factor that will govern the extent to which C-H activation–and other catalytic methods–gain acceptance as generalizable routes to construct molecular complexity.

**Scheme 3:**
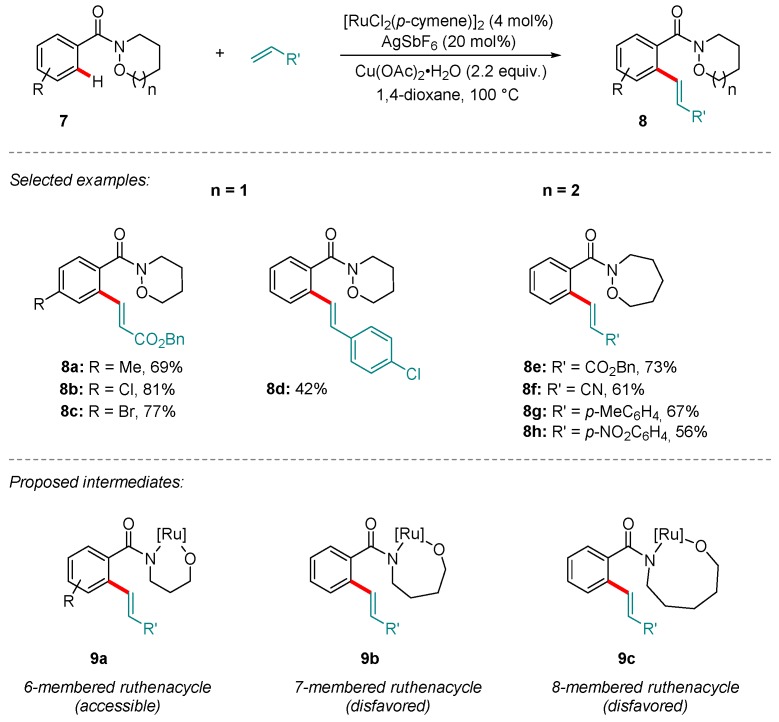
Ru-catalyzed C-H olefination using directed by cyclic Weinreb amides without N-O bond cleavage. Putative intermediates **9a–c** are suggested to arise from the insertion of Ru into the substrate N-O bond.

Das and Kapur reported that five-membered cyclic amides of the type **7** (*n* = 0) typically underwent N-O bond cleavage, affording ring-opened products. To prevent this, and thereby retain the Weinreb amide functionality, they subsequently sought to show that N-O bond cleavage could be prevented through judicious substrate design [42]. Increasing the Weinreb amide size to six- and seven-membered rings (**7**, *n* = 1 or 2, respectively) rendered the N-O bond cleavage energetically unfavorable. Under otherwise unchanged conditions, the oxidative C-H olefination afforded products **8** (Scheme 3), restoring Cu(II) to its role as the terminal oxidant. Further manipulation of the cyclic Weinreb amide moieties in **8**, for example in their conversion to the corresponding aldehyde or ketones, was demonstrated to proceed in high yield.

An explanation for the greater relative stability of the 6- and 7-membered cyclic Weinreb amide groups in this protocol was proposed: In the course of the reaction, oxidative addition of the Ru center to the N-O bond would furnish intermediates of type **9**. Of these, only the formation of **9a** would be favorable; the 7- and 8-membered ruthenacycles (**9b** and **9c**, respectively) are presumably too high in energy to be accessed.

## 3. Co-catalyzed Reactions

The expense and eventual scarcity of second- (4d) and third-row (5d) transition metals has, in recent years, motivated a shift towards exploring the potential of their more abundant first-row (3d) cousins for catalytic C-H functionalization [43]. Cobalt stands out in this context as a comparatively cheap, abundant option that comes with a proven track record in broad areas of homogenous catalysis, including several industrially important reactions [44,45,46].

**Scheme 4:**
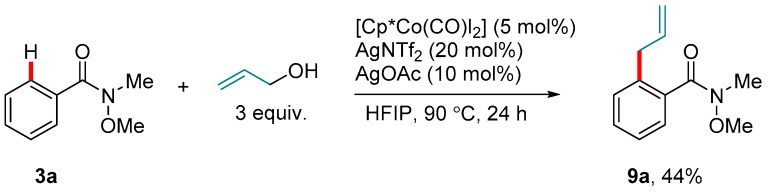
An initial example of the Weinreb amide-directed Co(III)-catalyzed *ortho*-C-H allylation.

In 2013, Yoshino and Matsunaga disclosed the first C-H *ortho*-directed functionalization catalyzed by Cp*Co(III) species [47,48]. In a subsequent study on the Co-catalyzed C-H allylation of aryl purines and benzamides using allylic alcohol, they demonstrated the new reaction also on Weinreb amide **3a** [49]. Here, the bench-stable complex Cp*Co(CO)I_2_, which was previously shown by Matsunaga and Kanai to be highly efficient for indole C2-H amidation [50], served as the pre-catalyst (Scheme 4). Hexafluoroisopropanol (HFIP) [51,52] was needed for the C-H allylation, as were acetate salts. The authors interpreted the latter as an indication that the C-H activation proceeded via a Concerted-Metalation-Deprotonation (CMD) mechanism [53], as has been elucidated in more detail for second row transition metals [8,40,54]. The moderate yield of product **9a** in this reaction was attributed to the weaker coordinating ability of the Weinreb amide group compared to its simpler benzamide relatives or, indeed, to that of the purine N_sp_^2^ centers.

**Scheme 5:**
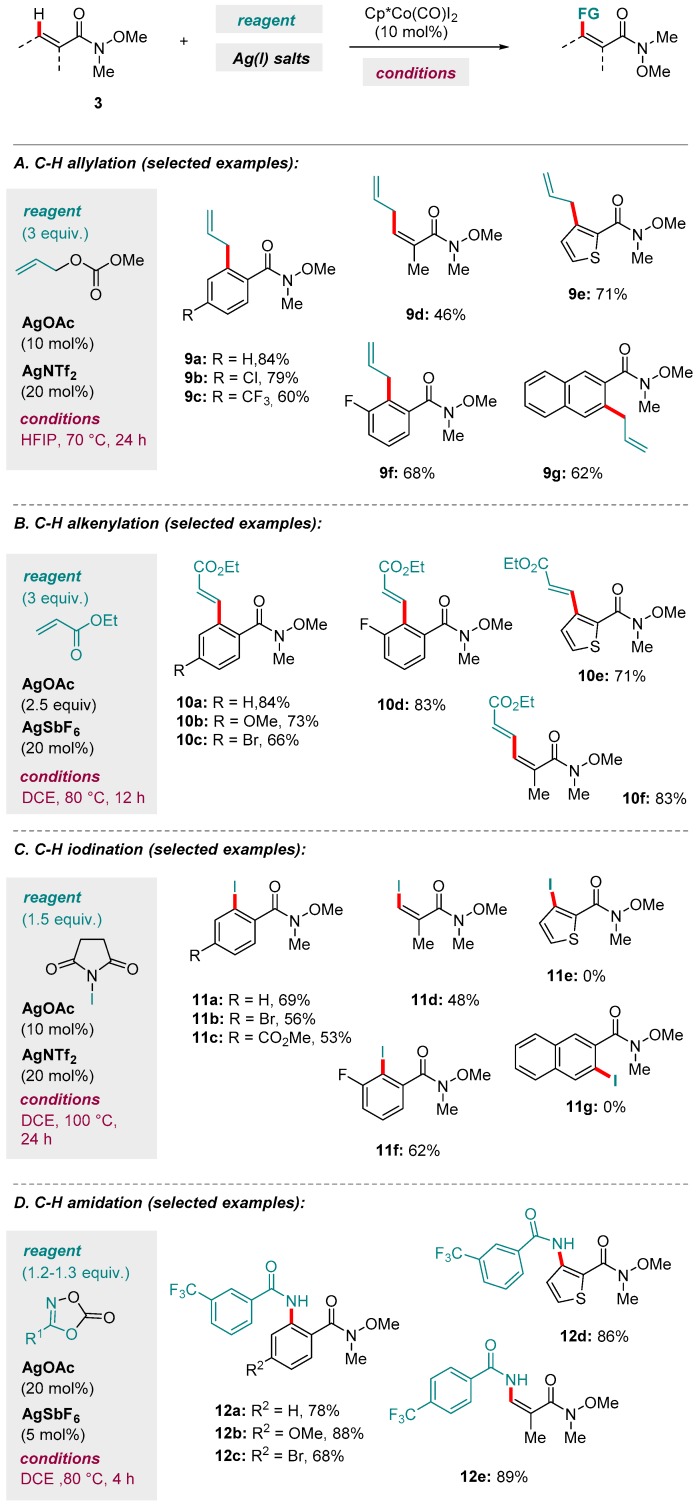
Selected examples of C-H transformations enabled by a Co(III)-catalyzed system.

**Figure 3:**
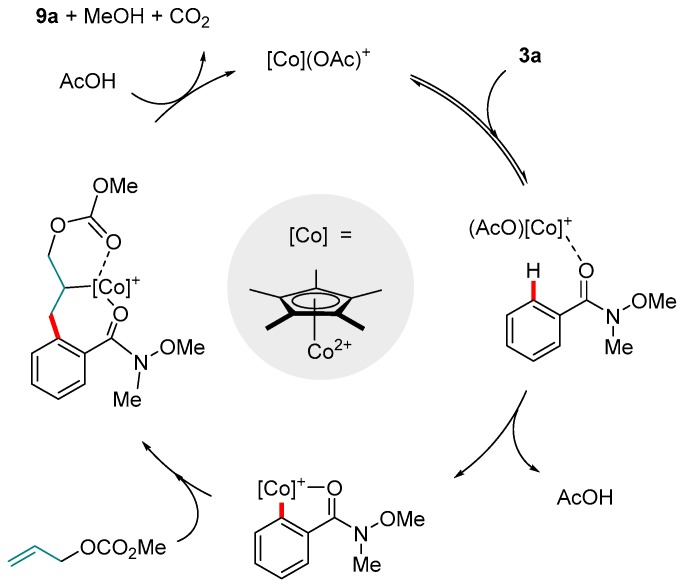
A mechanism proposed by Yoshino and Matsunaga for the Co(III)-catalyzed C-H allylation of Weinreb amides.

In a later study, the group published a considerably expanded range of Co-catalyzed C-H functionalizations using aromatic Weinreb amides (Scheme 5) [55]. These included C-H allylation using allylic carbonates (Scheme 5A), C-H alkenylation under oxidative conditions (Scheme 5B), C-H iodination using *N*-iodosuccinimide (Scheme 5C) [56] and C-H amidation using dioxazolones (Scheme 5D). These systems relied on similar combinations of [Cp*Co(CO)I_2_] pre-catalyst, a cationic silver additive (AgNTf_2_ or AgSbF_6_) and AgOAc. Unlike in the Ru-catalyzed case (see Scheme 2), the N-O bond of the Weinreb amide moiety was preserved. Competition and kinetic isotope exchange experiments showed that the C-H activation event in the case of the allylation reaction was both rate-limiting and all but irreversible, supplying further evidence for the CMD pathway Yoshino and Matsunaga proposed in their earlier study [49]. Their proposed mechanism for the Co(III)-catalyzed C-H allylation is shown in Figure 3. In related recent work, Whiteoak and Hamilton have elucidated the mechanistic details governing the oxidative Co-catalyzed C-H alkenylation of amides, and specifically whether and why alkyl or alkenyl products are obtained depending on the choice of alkene substrate [57].

The relevance to wider synthetic contexts of the transformations reported by Yoshino and Matsunaga was demonstrated through the conversion of the Weinreb amide group in their various products to the corresponding ketones, aldehydes and alkenes. This thus delivers several motifs whose preparation might otherwise be longer and more demanding.

## 4. Pd-catalyzed Reactions

Palladium is perhaps the most firmly established metal for coupling chemistry [58,59,60,61,62], and this has translated to its high level of popularity for C-H functionalization reactions [16,63,64,65,66]. Many Pd-based catalysts are well-defined [67,68], permitting systematic tuning of their properties, and are understood, most usually, to work via Pd(0)/Pd(II) or Pd(II)/Pd(IV) manifolds [62,64,66]. Despite the prevalence of many *ortho*-directing groups, including several closely related amides [30], there have been relatively few examples of Pd-catalyzed reactions using Weinreb amides.

**Scheme 6:**
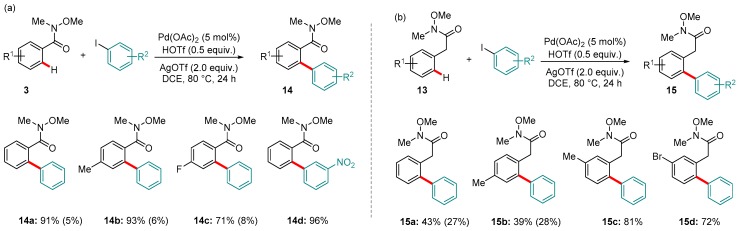
Pd-catalyzed C-H arylation of (**a**) aryl and (**b**) benzyl amides. Yields in parentheses refer to the amount of di-arylated products detected.

In 2015, Wang and co-workers reported the first Weinreb amide-directed C-H functionalization catalyzed by Pd. The reaction took place between either aryl Weinreb amides **3** (Scheme 6a) or benzyl amides **13** and iodoarenes using Pd(OAc)_2_ as the pre-catalyst in DCE solvent [69]. Both Weinreb types showed high tolerance towards electron-donating and electron-withdrawing substituents, as well as halogens (e.g., the bromide in **15d**). However, benzyl amides showed considerably worse mono-selectivity, except if *meta* substituents were present (e.g., **15c** and **15d**) presumably to impart steric hindrance.

**Figure 4:**
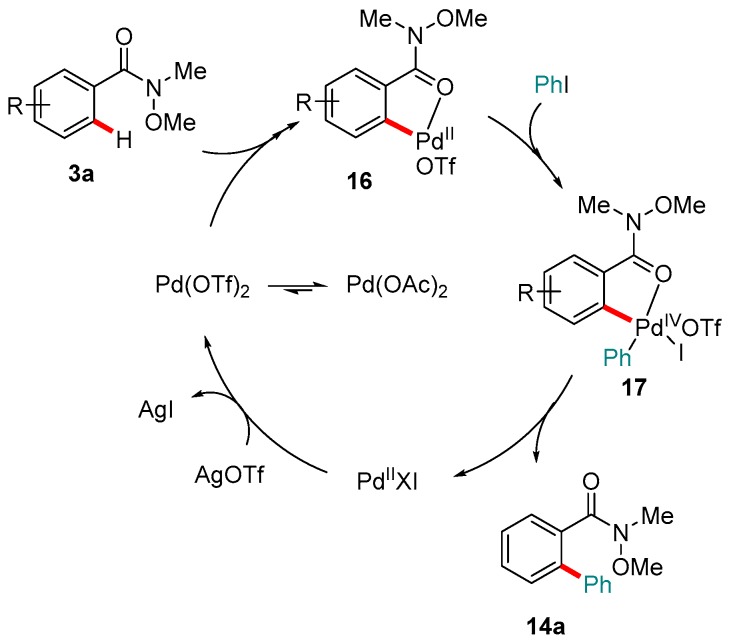
Mechanism proposed by Wang and co-workers for the C-H *ortho*-arylation of Weinreb amides proceeding via Pd(IV) intermediates.

Figure 4 shows one of the proposed mechanisms, adapted for the specific case of substrate **3a**. A kinetic isotope effect (KIE) of 1.1 suggested that the C-H activation step itself (forming **16** from **3**) was not rate-determining. Oxidative addition of the aryl iodide to **16** was postulated to form **17** from which the product **14a** is formed via reductive elimination. The authors reported that AgOTf, as well as acting as an oxidant, was crucial for the reaction and hypothesized that in situ generated Pd(OTf)_2_ is a key aspect of the catalytic cycle. (We note that the various roles Ag plays in Pd-catalyzed reactions have recently been reviewed by Pérez-Temprano and co-workers [70].)

**Scheme 7:**
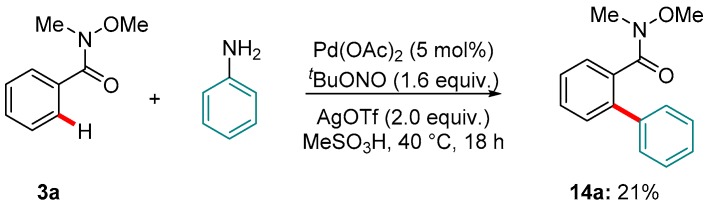
Bhanage’s C-H arylation using aniline as the electrophile source.

To the best of our knowledge, the only other example of a Pd-catalyzed Weinreb amide-directed C-H arylation comes from a single entry in a study by Bhanage and workers on the use of anilines as arylating reagents for N-methoxybenzamides (Scheme 7) [71]. Conditions were largely analogous to those described for Wang’s reaction above, with the principal exception that aniline underwent in situ oxidation using *^t^*BuONO to generate the electrophilic coupling partner. However, the yield for the Weinreb amide specifically was low, indicating that although this approach holds some promise, it is harder to optimize for the Weinreb amide compared to the using more conventional coupling partners.

**Scheme 8:**
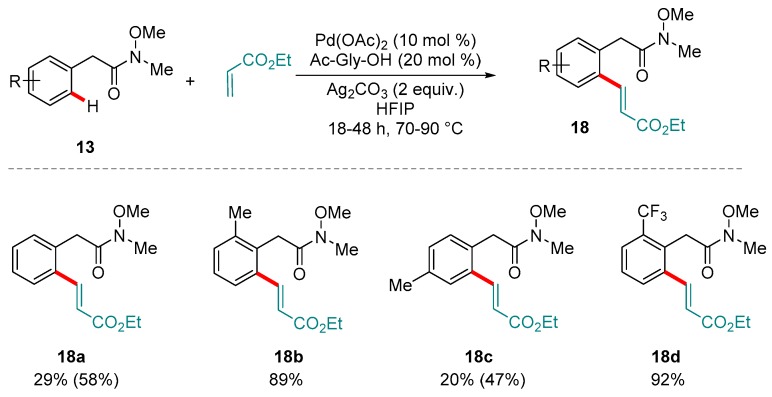
Yu and co-workers’ protocol for the oxidative *ortho* C-H acetoxylation of benzylic Weinreb amides. Yields in parentheses refer to the amount of di-olefinated products detected.

Also in 2015, Yu and co-workers described conditions for the efficient alkenylation and acetoxylation of Weinreb amides (Scheme 8) [72], overcoming the additional entropic penalty imposed by the extra methylene unit present in the directing group (and thus the challenge of forming seven-membered palladacyclic intermediates). In keeping with related protocols reported by Yu and co-workers, the presence of Ac-Gly-OH was found to be crucial to facilitate the C-H functionalization step [73].

Yu and co-workers found the olefination reaction to have moderate to excellent yields, exclusively mono C-H olefination with *ortho* and *meta*-substituted benzyl Weinreb amides, but reduced selectivity if the substituents were positioned *para* with respect to the directing group (Scheme 8).

**Scheme 9:**
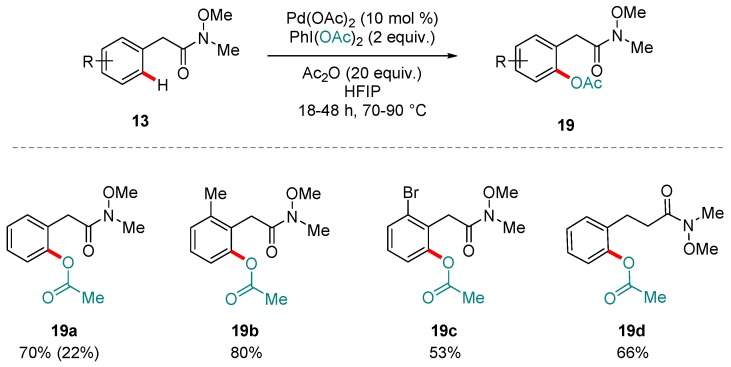
Yu and co-workers’ protocol for the oxidative *ortho* C-H acetoxylation of benzylic Weinreb amides. Yields in parentheses refer to the amount of di-acetoxylated products detected.

The acetoxylation of benzyl amides (Scheme 9) used PhI(OAc)_2_ as the oxidant in the presence of either Ac_2_O or tert-butyl peroxyacetate (MeC(O)OO*^t^*Bu). Yields and tolerance towards various substituents were good (examples **19a–c**). Furthermore, it was possible to expand the scope by an extra methylene unit (i.e., the reaction can proceed with good efficiency via an 8-membered palladacycle) to give product **19d**.

**Scheme 10:**
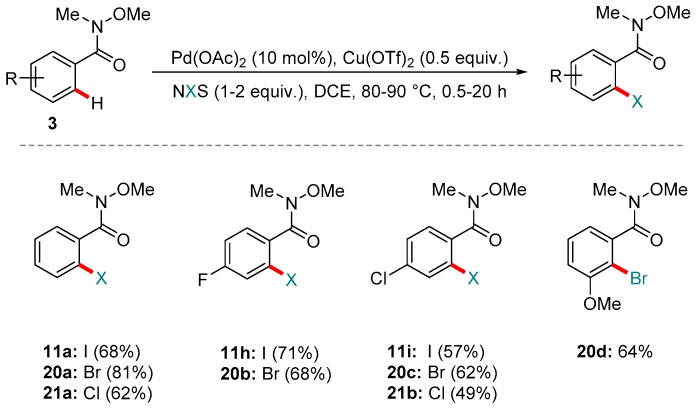
Kapur and co-workers’ reaction for the *ortho* C-H halogenation of aromatic Weinreb amides.

The introduction of halogens to organic substrates is an inherently valuable pursuit in methodology development, owing the great versatility of C-halogen bonds in synthesis [74]. In 2017, the group of Kapur disclosed the Pd-catalyzed C-H halogenation (iodination, bromination and chlorination) of Weinreb amides (Scheme 10) [75]. The reaction relied on a combination of Pd(OAc)_2_ and Cu(OTf)_2_ with N-halo-succinimide (NXS; X = I, Br or Cl) as the halogen source. The reaction worked well with a variety of substituents, except for nitro groups, which switch off the reaction from either of the *ortho* or *para* position.

**Figure 5:**
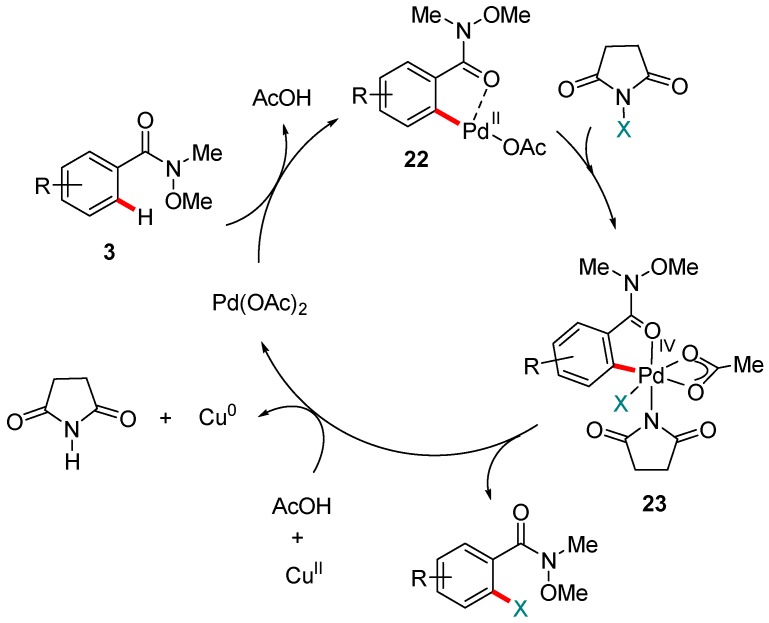
An adapted version of the mechanism proposed by Kapur and co-workers for the Pd-catalyzed *ortho* C-H halogenation of aromatic Weinreb amides. X = Cl^−^, Br^−^ or I^−^.

Mechanistic studies suggested that the C-H activation step was irreversible, but not rate-limiting. The catalytic cycle (Figure 5) was proposed to proceed via a route closely related to that of the corresponding arylation reaction (Figure 4, above): carbonyl directed cyclometallation to give **22** precedes oxidative addition into NXS, the latter of which produces Pd(IV) species **23** (a bimetallic Pd(III) species [76,77,78,79]—not shown—is also possible). Thereafter, reductive elimination releases the desired product.

**Scheme 11:**
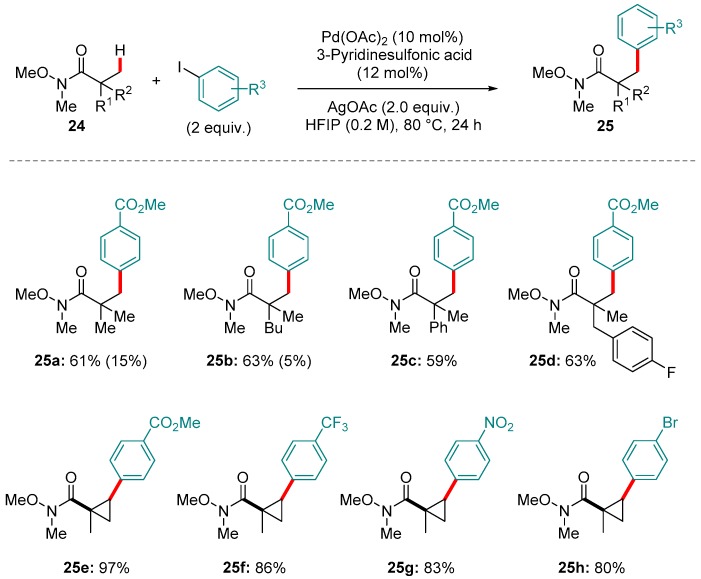
Selected scope from Yu and co-workers’ Pd-catalyzed C(sp^3^)-H arylation protocol enabled by Weinreb amide directing groups.

2018 saw Yu and co-workers extend the utility of the Weinreb amide directing group further [80]. This time, the Yu group used it as the basis for directing Pd-catalyzed C_sp_^3^-H arylation of alkyl groups (Scheme 11). The discovery of general and efficient methods that allow selective substitution of C(sp^3^)-H at transition metal centers is a long-standing challenge. For Pd, progress has been notably rapid in in the past few years [63,81,82,83], and its various aspects have been reviewed recently [65,84,85].

Yu and co-workers found that the inclusion of 3-pyridinesulfonic acid was crucial to the reaction, both Weinreb and other amides. Computational studies at the DFT level revealed that 3-pyridinesulfonic acid stabilizes cationic Pd intermediates during the reaction and that it promotes the dissociation of acetate ligands, which is required for the C(sp^3^)-H bond cleavage to occur [80].

## 5. Rh-catalyzed Reactions

**Scheme 12:**
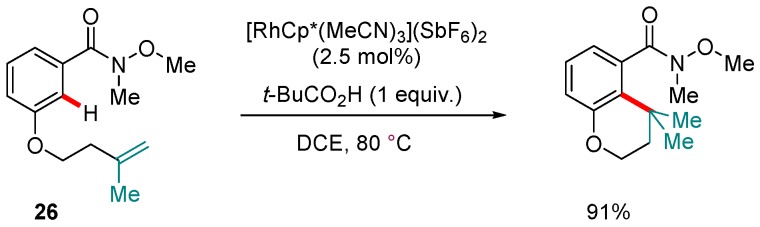
A Rh(III)-catalyzed, Weinreb amide-directed alkene hydroarylation.

The first two decades of this century have seen an explosion of interest in the use of Rh(III) catalysts for selective C-H functionalization. Substantial progress has been made in both scope [86,87] and attendant mechanistic understanding [7,88,89,90]. As part of their efforts to answer key questions relating to the regioselectivity of Rh(III)-catalyzed transformations, Rovis and co-workers reported in 2013 an intramolecular alkene hydroarylation directed by Weinreb amide **26** in excellent yield (Scheme 12) [91].

**Scheme 13:**
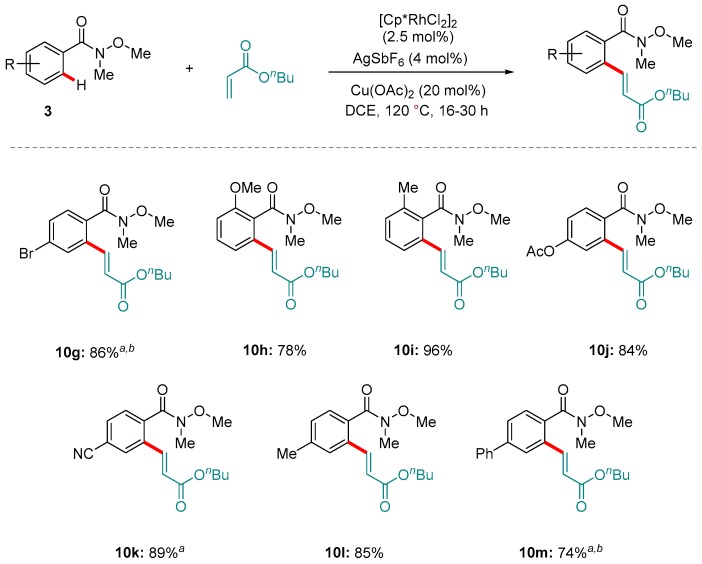
Selected examples from Wang and co-workers’ oxidative Rh-catalyzed C-H alkenylation directed by Weinreb amides. ^a^ [Cp*RhCl_2_]_2_ (2.5 mol%) and AgSbF_6_ (10 mol%) loadings were used. ^b^ A reaction temperature of 130 °C was used.

Wang and co-workers reported a closely-related but more elaborate system, involving Cu(OAc)_2_ as a catalytic oxidant regenerated under air to retain olefin functionality at the end of a cycle coupling aromatic Weinreb amides with alkenes (Scheme 13) [92]. An ample scope demonstrated the reaction’s tolerance towards various functional groups, including halogens, various *ortho*-substituents and a range of electron-donating and electron-withdrawing groups. Competition experiments revealed that electron-rich arenes reacted faster than their electron-poor counterparts.

**Figure 6:**
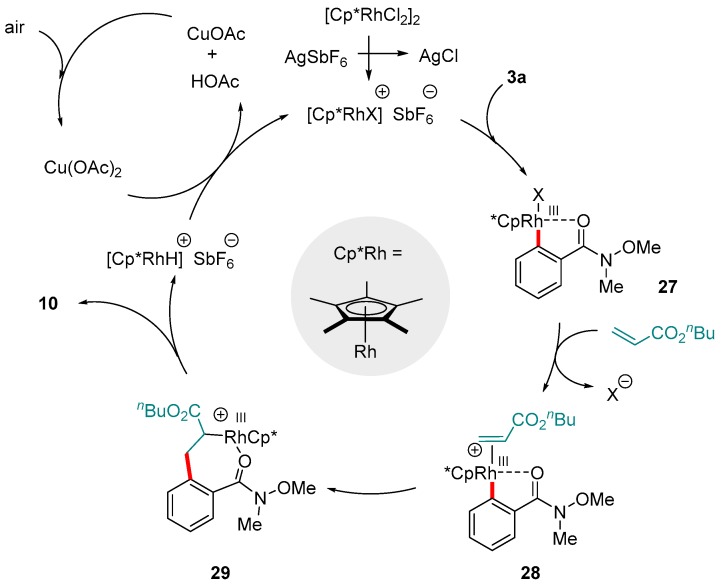
An adapted version of the mechanism proposed by Wang and co-workers for the oxidative C-H alkenylation of aromatic Weinreb amides catalyzed by Rh(III).

The mechanism proposed by Wang and co-workers (Figure 6) involved coordination of the Rh(III) center to the carbonyl moiety of the Weinreb amide, insertion of the alkene to the rhodacyclic intermediate (**27**), β-hydride elimination and regeneration of the active catalyst by Cu(OAc)_2_.

Whilst, strictly speaking, it represents a slight departure from the current focus on the Weinreb amide group, it is noteworthy that a very closely related class of substrates, has been used extensively wherein their C(O)NH-OMe bond as an “internal oxidant” for transition metal centers. Thus, C-H functionalization reactions may be performed without exogenous oxidants. This strategy has worked with a number of transition metals, Rh(I/III) catalytic cycles have dominated in this area [41].

In 2018, Qin and co-workers disclosed a near identical set of conditions to those used by Wang above, but using ethenesulfonyl fluoride (ESF) as the alkene coupling partner and 1,4-dioxane as the solvent [93]. However, the Qin group further observed that increasing the loading of AgSbF_6_ to 1 equivalent favored the formation of cyclic lactones. Residual water introduced from the hygroscopic AgSbF_6_ was proposed to promote the in situ hydrolysis of the Weinreb amide group to account for the lactone formation, though mechanistic work proved inconclusive. In switching away from Weinreb amides to N-methoxybenazmides (ArC(O)NH-OMe), Qin and co-workers were able to cause a similar oxidative cyclization involving insertion of the ESF double bond into the amide N-H unit.

**Scheme 14:**
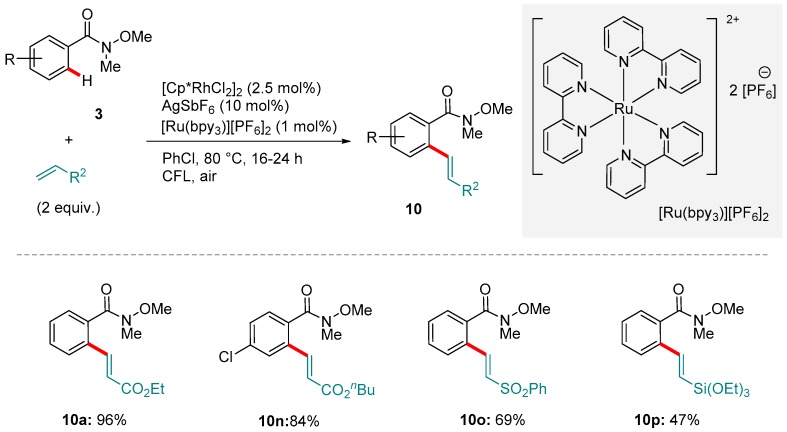
Rh-catalyzed olefination of aromatic Weinreb amides enabled by Ru photocatalysis.

Recent years have witnessed the rediscovery and subsequent explosion of interest in functionalizations enabled by photoredox catalysis. The photoexcitation of transition metal complexes serves as a greener and more economically viable method of generating radical intermediates able to initiate a wide range of valuable reactions [94,95,96,97,98]. One application of this is the replacement of terminal oxidants with lighter loadings of a photocatalyst whose oxidative power is obtained via photoexcitation. Rueping and co-workers applied this strategy to demonstrate that the oxidative alkenylation of aromatic amides, including Weinreb amides, is viable using a manifold analogous to that described by Wang’s group (see above), but driven by a visible light-regenerated Ru-based photocatalyst, [Ru(bpy)_3_][PF_6_]_2_ (Scheme 14) [99,100]. The protocol is notable not just for its efficiency and high functional group tolerance, but also for the fact that it could be extended to other amides as well as a variety of olefin substrates (various groups R^2^).

**Figure 7:**
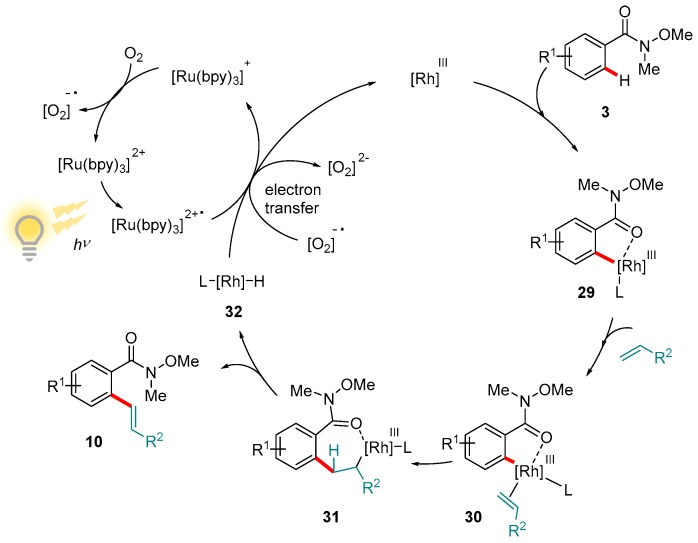
Mechanism proposed by Rueping and co-workers for the oxidative C-H olefination of aromatic Weinreb amides catalyzed by Rh(III) in the presence of a Ru photocatalyst.

The catalytic cycle proposed by Rueping and co-workers closely resembles to that of Wang: *ortho*-rhodation of **3** to give **29** coordination and insertion of the olefin (complex **30** to **31**) and *β*-hydride elimination to form the hydride intermediate **32** and the product (Figure 7). Reductive loss of the hydride and re-oxidation to Rh(III), however, is mediated by the photo-excited [Ru(bpy)3]^2+∙^ species, of which only a 1 mol% loading is required.

## 6. Ir-catalyzed Reactions

Boronates rank amongst the most versatile of groups; they may be converted to an extraordinarily broad range of functionality through a many different mechanisms [101,102,103]. Moreover, recent years have seen boronate-based methodology emerge as the basis of automated synthesis, which holds enormous potential to streamline the synthesis of many complex (hetero)aromatic and olefinic molecules [104,105,106,107]. Such advantages render especially important methods that allow the regioselective introduction of boronates to organic substrates. Amongst these, Ir-catalyzed C-H borylation ranks as one of the mildest and most enabling, as it is amenable to regiocontrol through sterics [108,109], directing groups (both to the *ortho* [110,111,112,113,114,115] and *para* [116] positions) and/or the inherent electronic properties [117,118,119] of a substrate.

**Scheme 15:**
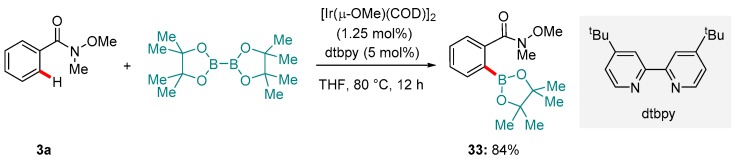
Ir-catalyzed *ortho*-C-H borylation of an arene using the Weinreb amide as a directing group.

Krska, Maleczka and Smith have demonstrated [120] that Weinreb amides rank amongst groups that competently direct Ir catalysts towards *ortho*-C-H borylation (Scheme 15). Although only a single entry using a Weinreb amide was reported in their Communication, the success of the reaction (a high yield and regioselectivity) was representative, suggesting that other advantages of the method, such as the high functional group tolerance, could easily be paired with the Weinreb amide functionality. Conditions for this transformation deviated little from those commonly used for Ir-catalyzed C-H borylation more generally: [Ir(*μ*-OMe)(COD)]_2_ as the pre-catalyst with dtbtpy as a ligand and B_2_(pin)_2_ as the boron source. The corresponding mono-borylated product, **33** was reported to form in 84% yield.

**Scheme 16:**
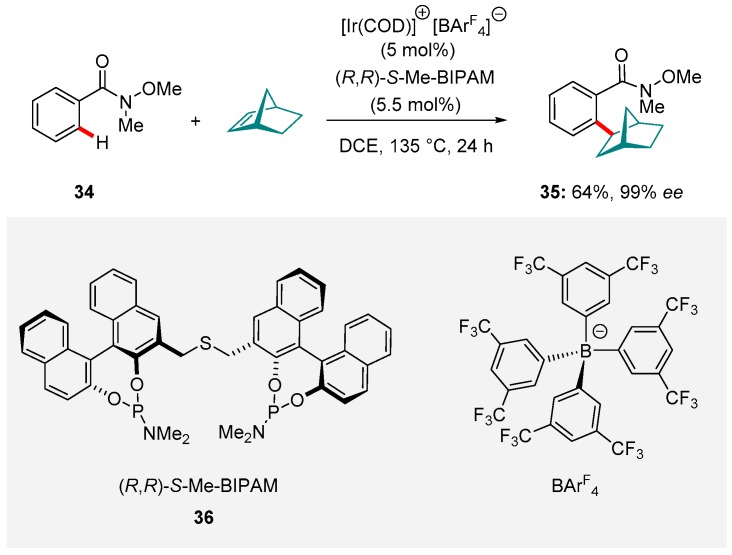
Ir-catalyzed enantioselective C-H hydroarylation of an olefin, directed by a Weinreb amide.

Enantioselective C-H functionalization marries the benefits of directly substituting a C-H bond with with establishing a new stereocenter in the product. This pursuit continues to inspire a growing body of research [121]. In 2015, Yamamoto and Shirai described an Ir-catalyzed protocol for the asymmetric intermolecular hydroarylation of arenes directed by oxygen based directing groups [122]. Although the scope included only a single example exploiting a Weinreb amide directing group (the transformation of **34** to **35**), this entry was representative both in terms of yield and selectivity (Scheme 16). Enantioselectivity was induced using the bidentate bis(phosphoramidite) ligand (*R*,*R*)-*S*-Me-BIPAM ligand (**36**) and the N-O bond was preserved in the product.

**Scheme 17:**
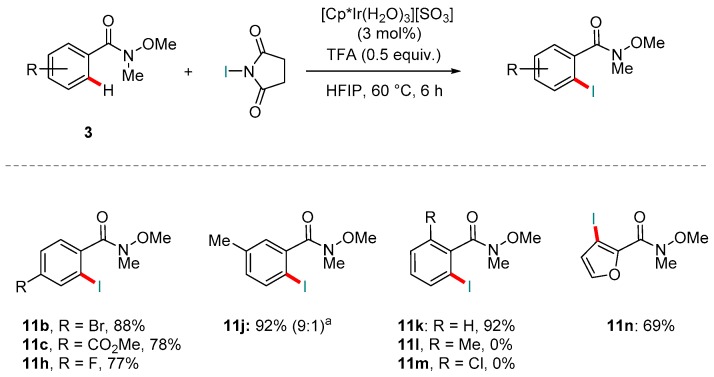
Ir-catalyzed C-H iodination of Weinreb amides developed by the Martín-Matute group. ^a^ Ratio of isomers is indicated in parenthesis; the major isomer is shown.

Most recently, the group of Martín-Matute has developed an Ir(III)-catalyzed C-H *ortho*-iodination of various amides [123]. The scope includes a strong focus on Weinreb amides. Their reaction used [Cp*Ir(H_2_O)_3_][SO_4_] as the catalyst precursor, and *N*-iodo-succinimide (NIS) as the halogenating reagent in the presence of trifluoroacetic acid. Selected examples are shown below (Scheme 17). It is notable that Weinreb amides returned only the mono-iodinated products **11**; a result only tertiary amides gave (primary and secondary amides gave at least small amounts of di-iodination). Whilst the substrate scope included a broad range of functional groups, substituents positioned *ortho* to the directing group at the outset restricted reactivity, presumably by imposing prohibitive amounts of steric hindrance.

The authors also performed a robustness screen [124,125] to identify the reaction’s tolerance towards various functional groups. To this end, various small molecules with different functional groups were added to the reaction mixture to see how the reaction would be affected. Tolerance towards several common functional groups, including ketones amides, carboxylic acids and alkyl halides proved excellent. Aldehydes and alkenes returned less satisfactory results, however. Mechanistically, the reaction was proposed to follow a pathway closely related to that of Martín-Matute and co-workers’ recently published Ir-catalyzed C-H *ortho*-iodination of aryl carboxylic acids [126]. The acid additive is understood to play a dual role: 1) activation of NIS via protonation of its carbonyl group and 2) by encouraging the dissociation of the iodinated product from the Ir center. Indeed, Martín-Matute and co-workers found in their optimization study on amides that lowering the amount of acid additive favored the formation of di-iodinated products for non-tertiary amides.

## 7. Conclusions

The Weinreb amide is a privileged functional group in organic synthesis that enables otherwise impossible transformations. Recent developments have seen C-H functionalization methodology greatly expand the range of chemical contexts in which its advantages may be exploited. Challenges remain, however. Presently, Weinreb amides are rarely explored as a substrate class in their own right; they are most often presented as specialized examples in studies describing a more general scope, typically amidst other amides. Thus, it is usual that only the simplest examples of Weinreb amides are demonstrated to work under newly developed reaction conditions. This is at least a little unfair since, as some of the examples described above demonstrate, Weinreb amides may return different results to other amides, for example by virtue of the reactivity of their N-O bond. Weinreb amides might, in this sense, be considered hitherto as “sleeper” substrates.

Despite this, it is evident that Weinreb amides can direct a wide range of C-H functionalization reactions catalyzed by transition metals. C-H functionalization, and catalytic methodology in general, is undergoing a shift of emphasis towards the use of less “endangered” [127,128], especially first-row transition elements for catalysis [43]. However, their use with Weinreb amides is rare. For example, to the best of our knowledge, no Weinreb amide-directed C-H functionalization reactions catalyzed by Mn, Fe or Ni have yet been reported.

Finally, it is unfortunate that the preponderance of conditions used for Weinreb amide directed C-H functionalization involve toxic halogenated solvents. Increasing legislative pressure is being brought to bear on this problem, with a particular focus against 1,2-dichloroethane (DCE) [129], which features in many of the reactions discussed above. We look forward to the use of Weinreb amides under greener conditions [130,131,132].
